# Sexual femicide, non-sexual femicide and rape: Where do the differences lie? A continuum in a pattern of violence against women

**DOI:** 10.3389/fpsyg.2022.957327

**Published:** 2022-11-01

**Authors:** Georgia Zara, Sarah Gino, Sara Veggi, Franco Freilone

**Affiliations:** ^1^Department of Psychology, University of Turin, Turin, Italy; ^2^Department of Health Sciences, University of Piemonte Orientale, Novara, Italy

**Keywords:** violence against women, sexual femicide, non-sexual femicide, intimate partner violence, rape

## Abstract

Violence against women is a growing health problem, especially when perpetrated in intimate relationships. Despite increasing attention, there is little comparative evidence on the different types of violence involved and there is a paucity of research on sexual femicides. This study examines cases of violence against women in northern Italy, focusing on sexual and non-sexual femicides and comparing them with rape that does not result in femicides. The sample included 500 women who were victims of sexual and non-sexual femicides, and of rape. Results show sexual femicides mostly involved unknown victims or women who were prostitutes. Sexual femicidal offenders used improper weapons to kill their victims, acted in secluded locations, and fled the crime scene; their crime was more likely the result of predatory intentions, with antisociality and sexual deviance being the most significant factors related to this type of femicide. The criminal and violent pattern that characterized sexual femicides in this study shared significant similarities with the pattern of violence involved in rape. Rape victims were in fact mostly unknown, or involved in a brief relationship with their killer. When the victim was known it was more likely that the abuse occurred at home and in front of the woman’s children. Rapists were often under the effect of alcohol or drugs. Non-sexual femicides mainly involved known victims, and they were more often committed in the context of domestic disputes. It was not seldom that the long relationship between the victim and perpetrator was likely to be characterized by contentiousness, suggesting that the woman was often victim of an oppressive climate of emotional tension and domination. Morbid jealousy contributed to aggravating the tone of a controlling relationship. Non-sexual femicides bore more similarities to cases of rape within the pattern of intimate partner violence. Findings are discussed in terms of their implications for prevention and intervention.

## Introduction

A major public health problem and human rights violation plaguing humanity is violence against women (hereafter abbreviated as VaW) (World Health Organization [WHO], 2017). VaW takes different forms (emotional, sexual, psychological, physical, economic, and cultural) that vary in frequency, intensity, and severity (Marks et al., 2020). It also differs according to types (known or strangers) and number (single or multiple) of victims involved (Zara et al., 2019, 2021) and the nature of the relationships (intimate, familiar, acquaintance or superficial vs. no relationship) between victims and perpetrators (Gino et al., 2019). It is not uncommon for victims to be in serious danger of losing their lives (Matias et al., 2020) i.e., femicide. Femicide is a violation of the basic human right to life, liberty, and personal security (Russell and Harmes, 2001).

Despite a lack of reliable data making it difficult to estimate the true extent of VaW aggravating into femicide, the most consistent research finding across the world is that only a small proportion of femicide occurs within an anonymous setting (Zara and Gino, 2018). According to the United Nations Office on Drugs and Crime (UNODC, 2021), on average, every 11 min a woman is killed by someone she knows; often it is someone in her own family, someone with whom the victim had an affective and/or intimate relationship. In most cases, such a killing is the culmination of previous experiences of various forms of violence, including sexual violence, perpetrated against them.

The sexual component of violence against women (e.g., rape) deserves special attention in so far it would lead to a more comprehensive understanding of those cases in which women are predominantly raped but not killed, those cases in which the rape occurs later and in a context of an oppressive, abusive and domineering relationship, and those cases in which women are also killed before, during or after being sexually abused. Notwithstanding that literature on femicide has grown in recent years, specialized studies on sexual femicide are still limited and within the general conceptual framework of sexual homicide (Chan, 2017).

According to the United Nations, VaW is one of the least prosecuted and punished crimes in the world. Although it is extremely difficult to compartmentalize violence into discrete events, an understanding of the psycho-criminological factors that anticipate the occurrence of femicide (sexual and non-sexual) and of rape is essential for scientific and clinical reasons, but above all for preventive purposes, that is, also to prevent the pejorative transformation of violence into femicide. The multidimensional offensive pattern of rape includes sexual violence that is intertwined with intimate partner violence (rape within IPV) (Campbell et al., 2003; Spencer and Stith, 2020) or sexual violence *per se* (rape ‘only’) (Veggi et al., 2021).

The following pages describe these forms of violence against women: sexual and non-sexual femicides, each compared with rape.

At the first level of analysis, the primary focus brings together some of the worst and most extreme aspects of VaW by examining whether and to what extent sexual and non-sexual femicides are similar or different, and whether they share more similarities or differences with rape.

At the second level of analysis, the focus is more specifically on sexual and non-sexual femicides compared with rape that occurs within IPV and rape ‘only.’

## The shadow of sexual violence

Sexuality is the most intimate dimension of life (Zara, 2018) and its respect is paramount for the well-being of the person. It follows that the violation of sexuality is one of the most offensive forms of violence against a woman with long-term consequences upon her psychological, relational and physical health. It offends her as a person. It mortifies her sense of *self* because it deprives the victim of any choices of when, how, and with whom to share her search for intimacy and for pleasure (Tarzia, 2021).

Clinical findings on women who survived IPV show that abused women are rarely keen to share sexually abusive incidents unless specifically asked about them (Heron et al., 2022). According to Walker (1994, 2009), it seems that the sexual component of the abuse experienced by women is recounted as the worst in terms of both humiliation and physical pain. These findings are in line with what partially emerges (or cannot emerge) from statistics^1^ : the dark number behind rape and sexual assault is extremely high and this is testified by their low report rates (Truman and Morgan, 2018). Unacknowledged rape is a quite common phenomenon not only among college women (Littleton et al., 2007) but among women survivors in the community (Harned, 2005), who do not label their experiences as rape but instead use more benign labels, such as «miscommunication» or «bad sex» (Wilson and Miller, 2016). The *sexual script* (Masters et al., 2013) adopted in such circumstances acts as a copying mechanism, mostly soon after the event, when confusion is still the main sensation that obliterates awareness of what had really happened (Kahn et al., 2003).

Studies show that unacknowledged rape is more likely to occur when the perpetrator is known, often in an intimate and romantic relationship with the woman (Littleton et al., 2018), in a setting in which abuse of alcohol or binge drinking has altered any sense of control (Peterson and Muehlenhard, 2011), and in which the woman often declares that she did not engage in a clear resistance strategy (Cleere and Lynn, 2013). Taken together these findings reveal that a sexualized offense is experienced as an amplified trauma in which not a single part of the victim was left untouched. Studies also suggest that when rape occurs within a relationship it is more likely that it is embedded in a broader pattern of abuse and violence (Coker et al., 2000; Matias et al., 2020; Sardinha et al., 2022). Rape that escalates into femicide, i.e., sexual femicide, is a type of VaW of a relatively rare occurrence (World Health Organization [WHO], 2012). However, its impact is extremely destructive.

## Sexual and non-sexual femicides

### Sexual femicides

If the term femicide refers to the killing of women by men *because* they are female, the term sexual femicide refers to the extreme form of control in which sex is used to degrade them to the point of death. The nature of the relationship between victim and perpetrator tells *how* and *why* the victim is killed. Table 1 presents a description of all these forms of violence that are the objects of this study.

**TABLE 1:** Variables coding schema.

Variables	Definitions for coding protocol
Criminal careers	With the concept of ‘criminal careers’ is meant here the official crimes that led to convictions attributed to the individual perpetrator, as indicated in the forensic files examined, and the self-reported offenses as they are reported in the criminological and clinical files. We are aware that this is only a partial perspective of what a criminal career is. Albeit scientifically important, the study of criminal careers of sexual and non-sexual femicide perpetrators was beyond the scope of this study. For further details on the criminal career paradigm, see the specialized literature (Piquero et al., 2007; Zara and Farrington, 2016).
Femicide	The killing of a woman *because* she is a woman (Russell and Harmes, 2001). If the target of the violence is a specific woman it is likely that an Intimate Partner Femicide takes place (see below) (Dawson and Gartner, 1998; Gino et al., 2019; Zara et al., 2019).
Intimate Partner Femicide (IPF)	The killing of a woman by a man with whom she had an intimate relationship: husband, ex-husband, life partner, boyfriend, ex-boyfriend, lover, or person with whom she had a child. This also includes the situation where a man murders a girlfriend or acquaintance who refuses to engage in an intimate (emotional and/or sexual) relationship with him (Gellerman and Suddath, 2005). IPF explains *why* and *how* that targeted woman is killed (Campbell et al., 2007).
Sexual Femicide	This is a case in which the death of the woman consists of two main components: femicide and the sexual behavior of the perpetrator. This may involve repressed or displaced anger and sadism (Karakasi et al., 2017). Sexual behavior can occur before, during, and/or after the femicide. This leads to a further distinction: sexualized femicide or grievance femicide (see below), although in some cases there may be an overlap.
Sexualized Femicide	It is functionally related to the sexual element of the offense. In other words, the sexual aspect and the killing (femicide) are closely related. In sexualized femicides, the victims are often unknown (Beauregard and Proulx, 2002; Beech et al., 2005), although perpetrators may have targeted their victims in advance for a specific reason (e.g., prostitutes). Aspects such as sexual fantasies, sexual arousal, masturbation, actual penetration may even be expressed symbolically through further exploitation of the victim’s body or genital mutilation (Myers et al., 1999; Meloy, 2000).
Grievance Femicide	It is driven by high emotionality, resentment, and rage schemas in which concern about losing contact with the woman (e.g., fear of being abandoned, deserted or dismissed) in most cases, fear of losing control over her, is expressed through sexualized aggression (Johnson et al., 2017).
Rape	It encompasses sexual violence characterized mainly by aggressive sexualized violence against the victim. Specifically, it can consist either of rape ‘only’ or rape within Intimate Partner Violence (IPV), where the sexual violence is part of a broader pattern of abuse and assault in the context of (mainly) intimate violence (Bagwell-Gray et al., 2015; Veggi et al., 2021).
Stalking	It implies an obsessive pursue of intimacy, and it is often referred to as a psychosexual obsession. The victim is the focus of stalking. Previous relationships between the victim and stalker have an impact on how the obsessive behavior is framed (Fox et al., 2011). If they had an enduring relationship, it is more likely that the stalker’s emotional involvement in the relationship will be stronger (Douglas and Dutton, 2001). In some cases, victims could be strangers. These victims are unaware of any prior contact with their stalker, who may be motivated by a desire for an intimate relationship with the victim or sexual motives. Those targeted by stranger stalkers may be chosen for their physical and sexual attractiveness, social status, vindictive reasons, persecutory thinking, or a fantasized version of the victim. Meloy (1997b) proposed a rating system for stalkers based on acquaintanceship: those who were previously known, those who were previously sexually intimate, and those who were strangers.
**Types of relationship (between victim and perpetrator):** – Known – Unknown	According to the victimology literature, a victim is considered “known” if victim and perpetrator knew each other for at least more than 24 h prior to the femicide. A victim is considered “unknown or stranger” if the victim did not know the perpetrator (or vice versa) 24 h before the femicide.
**Intensity of relationship:** – Intimate – Superficial or Acquaintance	An intimate relationship involves an affective and sexual connection between two people which implies a sort of continuity. It is a form of relationship based on sporadic contact between two people or limited knowledge about each other.
**Quality of relationship:** – Contentiousness – Jealousy	It implies the presence of psychologically erosive, negative, intense, and enduring emotional strain between people in a relationship (Birkley and Eckhardt, 2015; Zara et al., 2019). Contentiousness can only be assessed if the victim and perpetrator know each other. It is an emotion that exposes individuals to extreme dangers, which is why it is considered a dangerous passion (Buss, 2000; Freilone et al., 2020). The dark side of jealousy (often referred to as morbid or pathological) can cause men to explode violently to reduce the likelihood of their partners straying, and exposes women to the risk of being killed. In this study, jealousy was coded as present if the forensic and clinical record mentioned that it was one of the factors identified to explain the violence perpetrated against the victim.
Prostitutes	Prostitutes are sex workers who provide sexual services as a labor or an income-generating activity (Adriaenssens et al., 2016; Dylewski and Prokop, 2021). Sex exchange for money is the object of the transaction between the prostitute, as a provider of a service, and the client. Prostitutes work either with their own client base (e.g., call girls, escorts, and streetwalkers) or on behalf of other individuals or industries that procure clients for them within a lucrative business (e.g., sex clubs) (McGuire and Gruter, 2003). Violence against prostitutes reifies a justification for violence against women and conveys the idea that prostitutes are at their client’s disposal (Salfati et al., 2008; Zara et al., 2021), making them more at risk of violence and femicide (Sorochinski and Salfati, 2019).
Motives for offending	Following the typologies available in the literature and in accordance with the precise information in the forensic files and pathologist’s reports, two macro-categories were developed. They distinguish (1) oppressive, domineering, and multi-problematic relationships in which victims were placed in a state of inability to disengage from the perpetrator (Johnson, 2008; Zara and Gino, 2018; Gino et al., 2019) and (2) predatory motives, sexual deviance, and antisociality (Petersson et al., 2019). Motives for offending involving mental disorders are of particular interest in psychological and clinical settings and in the criminal justice system (Zara, 2013). Mental disorders are, however, neither necessary nor sufficient causes of violence (Stuart, 2003). Research shows that serious mental illness of the perpetrator is rarely the cause of violence against women *per se*: when present it is more likely in cases of domestic killing (Oram et al., 2013; Sorrentino et al., 2020; Caman et al., 2022) rather than in cases of sexual offending and IPV (Kelley et al., 2020). Studies on the mental illness in sex offenders show that rates of psychotic diagnoses range from 5 to 10% (Fazel et al., 2007). In this study the mental disorder of the perpetrator involved only a small percentage of cases (see Results section).
Overkill	The definition of overkill employed in this study was bound to the idea of excessiveness of violence, of amount or severity of wounds, and of trauma beyond that necessary to cause death (Zara and Gino, 2018; Trojan et al., 2019; Zara et al., 2019). Overkill was present if the victim sustained multiple injuries within one or more causes of death, i.e., multiple gunshot (at least three) or a combination of multiple gunshot with stab wounds and/or mechanical asphyxia, and if the multiple wounds of the same type involved two or more different body regions and were considered causes of death in each. It is agreed that overkill expresses a rage that is not sated even by the death of the victim. When the victim is for the perpetrator “not dead enough,” overkill is likely to emerge.
Weapons	Proper weapons are defined as instruments conventionally designed for combat and intended to inflict physical harm. Firearms and stabbing weapons (e.g., pistols, guns, and knives) are classified as proper weapons. Improper weapons are instruments that are not specifically intended to injure or kill, but can be used to fatally injure a person. Examples of improper weapons include blunt objects (e.g., a stick, a screwdriver) (Pelletier and Pizarro, 2019).
Body Interference or Exploitation	It is any act directed against the victim that leads to further abuse of her body after or during the crime (e.g., using needles on the victim’s legs after battering; inserting wire into the victim’s body cavities). In some cases, the perpetrator derives gratification from inflicting severe suffering (as a form of torture), to perhaps manifest absolute control over the body of the victim, or killing the victim. In such cases, the exploitation of the body may begin on the living person and continue after death, or it may be practiced after death (Salfati and Taylor, 2017).
Body mutilation	Body mutilation is the deprivation of a limb or other body part or organ, or severe disfigurement (Dorland, 2012; Guggenheimer et al., 2021). It could be seen as offensive mutilation in order to humiliate the victim. Resentment and strong aggression or hatred may cause the aggressor to disfigure the face or genitals (Karger et al., 2000). It could also be seen as a way to facilitate the disposal of the body or to allow the body to be transported and hidden in a place where it would not be noticed and make identification difficult (Braulin, 2002; Di Nunno et al., 2006; Konopka et al., 2007).
Perpetrator’s Reactions after crime	The types of reactions of the perpetrator refers to the behavior following the commission of the offense. It is defined as ‘active’ when the perpetrator flees or attempts to (e.g., running away) or denies his responsibility for the crime. It is defined as ‘passive’ when the perpetrator confesses the crime or commits suicide soon after the offense.

There is a dearth of specific studies on sexual femicide, and most investigations focus on sexual homicide (Chan, 2017). The use of sexual violence in the form of rape is an extreme form of control against a woman and sexual femicide is considered the most misogynistic form of violence in which a woman is reduced to a sexual commodity or a mere object of control, who once sexually used, could be thrown away (Horley and Clarke, 2016). Therefore, sexual femicide becomes the criminogenic setting where VaW can be explored in its boldest form, because the final gesture of femicide combines, in the act of killing, sexualized aggression, contempt, entitlement, predatory attitudes, psychological and physical domination, and vindictive interests (Salfati and Taylor, 2017).

To understand what distinguishes lethal from non-lethal sexual assaults and whether sexual killers constitute a distinct group of sex offenders, Stefańska et al. (2015) compared sexual killers to sex offenders (specifically offenders involved in rape or attempted rape). Overall, these groups had more similarities than differences (Stefańska et al., 2015).

According to Beauregard and Martineau (2016), sexual murderers seem to have similar characteristics (combination of deviant sexuality and antisociality) to violent, non-homicidal sex offenders, defined as individuals involved in a heterogeneous criminal career (Merlone et al., 2016; Zara and Farrington, 2016) but also primarily preoccupied with sex (Zara et al., 2019).

As suggested by Carter and Hollin (2014), it might be relevant to place the offense in a situational context to understand whether the killing and the sexual element of the violence are directly or indirectly related. When they are directly linked, the act of killing is itself sexually gratifying, or the killing facilitates sexual acts on the victim’s body at the time or following the killing. When the killing is indirectly linked to rape, the killing is not a source of sexual arousal or stimulation but is an extreme reaction to restore control (e.g., the victim is killed because she is trying to escape or end the relationship).

Despite the rich attempts to establish a comprehensive set of criteria to define sexual killing (Ressler et al., 1988; Chan, 2015; Carter et al., 2017), little is known about what makes a femicide sexual and what makes femicidal sexual offenders distinct from non-femicidal sexual offenders. The latter could be a form of hate crime that requires specific scientific, social, cultural, and clinical attention.

Burgess et al. (1986) were among the first to attempt to classify sexual homicide and to distinguish sexual homicide from simply a homicide resulting from a sexual assault. They maintained that sexual homicides “result from one person killing another in the context of power, control, sexuality, and aggressive brutality” (p. 252). Häkkänen-Nyholm et al. (2009) emphasized that most of the victims of sexual homicides are female.

A widely used definition of sexual killing based on physical evidence at the crime scene or in forensic records is that of Ressler et al. (1988). For a killing to be considered sexually motivated, at least one of the following criteria must be met: (1) the victim lacks clothing; (2) the victim’s genitals are exposed; (3) the body was found in a sexually explicit position; (4) an object was inserted into the victim’s body cavity (anus, vagina, or mouth); (5) there is evidence of sexual intercourse (oral, vaginal or anal); (6) there is evidence of substitutive sexual activity (e.g., masturbation and ejaculation at the crime scene, or body exploitation) or sadistic sexual fantasies (e.g., genital mutilation).

Holmes and Holmes (2001), simplified the classification, and defined sexual homicide as the combination of lethal force with a sexual element.

Chan (2015, p. 7) added to the previous classification two new criteria: (1) a legally admissible admission by the offender regarding the sexual motive of the crime that intentionally or unintentionally results in homicide; (2) an indication of sexual elements of the crime from the offender’s personal effects (e.g., home computers and diary entries).

Salfati (2000) differentiated types of homicide based on their behavioral nature found at the scene, and on the dual model of aggression by Feshbach (1964): expressive and instrumental aggression. Expressive (or hostile) aggression typically occurs in response to circumstances that trigger anger, with the intention of making the victim suffer. In contrast, instrumental aggression results from a desire to acquire objects or status (e.g., valuable items, control, and territory) regardless of the cost. This distinction suggests that expressive aggression may more likely be exercised against known victims, whereas instrumental aggression may more likely be exercised against unknown victims.

### Non-sexual femicides

Non-sexual femicides is characterized by violence that escalates into a lethal epilog. Studies show that non-sexual femicide includes some or all of the following characteristics: threatened physical or sexual violence, economic and cultural violence, and emotional or psychological abuse perpetrated by a man (usually known) to the victim. In most cases, the perpetrator is a current or former intimate partner (Campbell et al., 2007; Neppl et al., 2019; Marco Francia, 2021; Zara et al., 2022). Perhaps, it is the shared history between victim and perpetrator that makes this type of violence escalate to femicide.

Skott et al. (2018) compared sexual femicides^2^ with non-sexual femicides in Scotland (United Kingdom). In their study, they examined data from a national police database and compared 89 male sexual femicidal offenders who had killed adult women with 306 male non-sexual femicidal offenders who had also killed adult women. The sexual femicides in their sample appeared to be more likely associated with instrumental aggression and sexual deviance, making sexual femicidal offenders more comparable to sex offenders than to other femicidal offenders.

In a study by Langevin et al. (1988) it was found that victims of non-sexual femicide tended to be older than the two sexual groups, and that sexual femicidal offenders were more likely to attack a female victim compared with non-sexual femicidal offenders. Examining sexual and non-sexual femicidal offenders they have found many differences between these criminal career groups (Langevin et al., 1988).

If sexual femicide differs from non-sexual femicide in terms of victims and perpetrators, this could have important implications not only for future research but also for policy, interventions, and rehabilitation programs for perpetrators.

Another study (Stefańska et al., 2020) suggests that sexual femicide could represent a hybrid offense combining rape and femicide. While rape may serve as a reminder of *sexual proprietariness* of a woman by a man (Wilson and Daly, 1993; Spencer and Stith, 2020), femicide may serve as the ultimate form of revenge for certain types of abusive and controlling personalities (Serran and Firestone, 2004). The combination of rape and femicide is the most prevalent form of sex-related killing (Geberth, 2016) intensified by the intimacy of the relationship (Geberth, 2018).

Given that several studies (Salfati and Canter, 1999; Salfati, 2000; Beauregard et al., 2018; Skott et al., 2018) have found more similarities than differences between sexual femicide offenders and rapists, it is plausible that sexual femicide should be considered an extreme variant of rape rather than a sexual variant of femicide.

Hence, according to Stefańska et al. (2021), understanding that hybrid should be the primary focus of psychological investigation.

## This study

This study examines cases of violence against women in northern Italy, focusing on sexual and non-sexual femicides and comparing them with rape that does not result in femicide.

The aim is threefold. Firstly, to explore similarities and differences in femicides: femicides that include sexualized violence and femicides that are not sexualized. Secondly, to analyze types of sexual femicides and to explore whether certain factors are more likely involved in either sexualized or grievance femicides. Thirdly, to compare cases in which rape is acted out without the escalation into femicide with cases in which rape is also a key part of the femicide dynamic. The interest is to see the extent to which femicides, sexual and non-sexual, in Italy involve the same risk and criminogenic factors of other forms of violence against women such as rape.

According to our knowledge, this is the first time a comparative study of different forms of VaW (non-sexual femicide vs. sexual femicide vs. rape) was conducted in Italy. While we are aware that violence against women is rarely a discrete event, especially when the victim knows the perpetrator and has a relationship with him, as shown in other studies (Flynn and Graham, 2010; Davies et al., 2015), this study sheds light on the seriousness of any forms of violence above and beyond what is considered the most dramatic aspect of it: death.

### Research questions

This study addresses the following questions:

(1) Are sexual and non-sexual femicides more similar or different in terms of victims, perpetrators, and characteristics of the exhibited violence?
(2) Are all sexual femicides of the same kind?
(3) What do sexual femicides have in common with rape?
(4) What do non-sexual femicides have in common with rape?

## Materials and methods

### Data collection and information for the study

The data used in this study were collected from a multisite project^3^.

The first phase of the study consisted in identifying the victims of femicide; information about age, profession, previous criminal records, prior involvement in violence, dynamics of the femicide; types of relationships between victims and perpetrators were also gathered.

Information on further exploitation of victims’ bodies and mutilation, as stated in the pathology reports, was collected. Forensic files from the Court of Turin were also examined, along with files from the archive of the medical experts who assessed the cases. This multisite study excluded all cases of women’s natural death or suicide.

In order to be able to explore differences between sexual femicides and rape that did not lead to the death of the victim, cases of sexual offending perpetrated by men against women, i.e., rape were examined.

### Variables

All information collected was classified according to the following dimensions: age, nationality and occupation of the victim and the perpetrator, location of the violence (e.g., at home: either in the victim’s or the perpetrator’s home; in a public place: usually in an isolated/secluded place in the outskirt of a city or town), medico-legal aspects of the murder (e.g., weapons used, substance abuse, i.e., alcohol and/or drugs, body mutilation and other forms of exploitation of the victim’s body), the perpetrator’s reaction after the crime (e.g., active vs. passive), the types of sexual femicides (e.g., sexualized femicide or grievance femicide). The type (known vs. unknown), intensity (intimate vs. acquaintance/superficial), duration of the relationship between victim and perpetrator (short- to medium-term vs. long-term), and the possible presence of children provided researchers with information to examine the context in which the crime occurred and to distinguish between sexual femicide, non-sexual femicide, and rape (see Table 1 for details).

In addition, contentiousness between victim and perpetrator and morbid jealousy were examined. These dimensions are relevant to assessing (1) the presence of violent incidents previously reported to the police, along with a pattern of frequent violent rows (contentiousness); (2) the dynamics of femicide and rape by examining the role of possessiveness, intrusive control, and obsessive desire for intimate exclusivity (morbid jealousy) in triggering the violence. In examining femicide, overkill (i.e., excessive force beyond what is necessary to cause death) was also considered.

### Data coding

Variables related to victims and perpetrators (such as age, nationality, and profession), locations of crime (e.g., indoor or outdoor), and the medico-legal aspects of the offense (e.g., body exploitation) were directly extrapolated from the scientific and forensic material available. Variables related to the violence dynamics were coded by two independent judges. Coding protocols used to formulate each specific variable are extremely relevant for reconstructing the data into a coherent framework, to explain the meaning of each variable endorsed and to differentiate them. Behavioral variables were dichotomized (see later for the rationale behind dichotomization) into either present or absent. This procedure identified 18 variables related to victim-perpetrator relationships, motive, and context of violence, as well as the variable “criminal career” that is here measured by criminal history recorded in the forensic file.

Regarding the quality of the relationship, contentiousness implies the presence of negative, intense, erosive and enduring emotional strain between people in a relationship (Birkley and Eckhardt, 2015). According to Jordan et al. (2010), overkill is described as the excessive use of force that goes further than what is necessary to kill. It involves multiple injuries and results in one or more causes of death or multiple wounds distributed over two or more regions of the body (Salfati, 2003; Trojan et al., 2019).

Table 1 summarizes the variables explored, accompanied by the coding definition.

Body exploitation implies interferences with the body of the victim, either when alive (as in the case of sex offending) or once dead (as in the case of femicide). In some cases, the perpetrator derives gratification (including sexual arousal and satisfaction) from inflicting severe suffering. In such cases, the exploitation of the body may begin on the living person and continue after death, or it may be practiced after death (see Table 1 for a detailed description).

Body mutilation was based on the pathologist reports and was assessed as present (1) or absent (0). In line with the medical literature (Guggenheimer et al., 2021), mutilation was defined as the deprivation of a limb or another body part or organ, or severe disfigurement. This could be seen as offensive mutilation and is likely done to humiliate the victim. Resentment and strong aggression or hatred may cause the aggressor to disfigure the face or genitals (Karger et al., 2000). In such cases it could happen that the victim is not killed.

Regarding motives for killing, two macro-categories were developed according to the typologies available in literature that distinguish an oppressive, domineering and multiproblematic relational condition (coded as 0) (Holtzworth-Munroe and Stuart, 1994; Johnson, 2008), and predatory motives, sexual deviance and antisociality (coded as 1) (Petersson et al., 2019).

In order to explore the extent to which sexuality was a key element of the killing when comparing victims, sexual femicides (Higgs et al., 2017) were operationalized into *grievance femicides* (coded as 0) and *sexualized femicides* (coded as 1). A femicide was coded as *grievance* when it was driven by high emotionality, resentment, and anger. A femicide was coded as *sexualized* when there was forensic evidence that it was functionally related to the sexual element of the offense (Beech et al., 2005) (see Table 1 for a detailed description).

Two independent raters carried out the categorization of data into the assessment of contentiousness, overkill, body exploitation, motives of crime, and grievance or sexualized femicide. Separate variables were created to indicate the presence (coded as 1) or absence (coded as 0) of assessed dimensions in each case. When a discrepancy emerged, the two independent raters discussed the case with the research group, and re-assessed it, until a better level of agreement was reached. The Cohen’s Kappa statistic (Cohen, 1960) provides a quantitative measure of the magnitude of agreement between observers that is corrected for chance, and it is appropriate for this type of data. The levels of agreement for the category of contentiousness (Cohen’s *K* was 0.98, *p* < 0.001), for the category ‘overkill’ (Cohen’s *K* was 0.98, *p* < 0.001), for the category of type of reaction by the perpetrator after the crime (Cohen’s *K* was 0.99, *p* < 0.001), for the category of type of body exploitation (Cohen’s *K* was 0.96, *p* < 0.001), for the category ‘motives of crime’ (Cohen’s *K* was 0.99, *p* = 0.001), for the category of sexual femicide (Cohen’s *K* was 0.97, *p* = 0.001) and for the categories of grievance and sexualized femicide (Cohen’s *K* was 0.98, *p* = 0.001), suggest a substantial inter-rater agreement coefficient for all of these variables (Viera and Garrett, 2005; McHugh, 2012).

## Analytical strategy

Descriptive and multivariate analyses with Odds Ratios (ORs) were carried out to explore specifically the characteristics of the sample involved and to provide an outlet for comparing: (1) sexual femicide and non-sexual femicide with rape; and (2) sexual femicide and non-sexual femicide with the subcomponents of rape: rape within IPV and rape ‘only.’

Specifically, 18 variables were examined and the OR was calculated to identify which of the 18 variables significantly and independently explained these types of violence. Also explored was whether the type of relationship between victim and perpetrator (known vs. unknown), and the intensity of the relationship (intimate vs. superficial/acquaintance) could affect the kind of violence the victim endured. The OR provides information about the existence, direction, and strength of an association between target and comparison groups regarding the likelihood of an event occurring (Farrington and Loeber, 2000). When ORs are higher than 1, situations characterized by that particular attribute have relatively higher odds of occurring than those that do not have that attribute (Zara and Farrington, 2020).

### Sample

**FIGURE 1:**
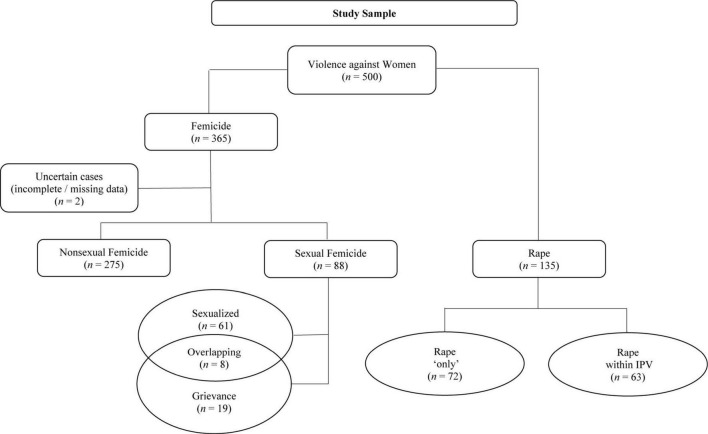
Flow diagram of different forms of violence against women involved in the study sample.

A total of 500 cases of VaW were selected and included in the sample. Specifically, the sample included 365 cases of femicide and 135 cases of rape. Figure 1 provides a detailed overview of the sample composition and distribution.

Perpetrators (*n* = 468) were on average 40.81 years old (*SD* = 15.65), mainly Italians (72.2%; *n* = 288) and employed at the time of the offense (64.2%; *n* = 235). Of them, more than a third had previous official criminal records (37.1%; *n* = 181). In only 6.1% of cases (*n* = 30) did the perpetrators suffer from a mental illness at the time of the crime, according to forensic psychiatric examinations.

In the present study, victims were also predominantly Italians (76.3%; *n* = 380) and professionally occupied (61.5%; *n* = 251). Victims were slightly older (*M* = 42.49 years old; *SD* = 20.16) than their perpetrators, but the difference was not significant, *t*(811) = 1.314, *p* = 0.09 (*d* = 0.09)^4^.

Table 2 summarizes the general information about the sample.

**TABLE 2:** Sample: Socio-demographic description of victims and perpetrators.

	Victims (*n* = 500)	
	*n*	*M* (*SD*) or %
**Victims**		
Age	439	42.49 (20.16)
**Nationality**		
Italian	380	76.3%
Foreigner	118	23.7%
**Profession**		
Employed	251	61.5%
Unemployed/Retired	157	38.5%

	**Perpetrators (*n* = 468)**	

**Perpetrators**		
Age	374	40.81 (15.65)
**Nationality**		
Italian	288	72.2%
Foreigner	111	27.8%
**Profession**		
Employed	235	64.2%
Unemployed	131	35.8%
**Previous criminal records**		
Yes	181	37.1%
No	307	62.9%
**Mental illness**		
Yes	30	6.1%
No	458	93.9%

Data regarding some variables were missing and this can explain why percentages do not always refer to the whole sample of 500 victims and 468 perpetrators studied.

Perpetrators of sexual femicide were on average significantly younger (*M* = 34.45 years old; *SD* = 11.22) than perpetrators of non-sexual femicide (*M* = 45.10 years old; *SD* = 17.22), *t*(256) = 4.01, *p* < 0.001 (*d* = 0.65), while no difference emerged when compared with perpetrators of rape (*M* = 35.78; *SD* = 11.14), *t*(179) = −0.70, *p* = 0.49 (*d* = 0.12). Perpetrators of rape were significantly younger than perpetrators of non-sexual femicide, *t*(345) = 5.59, *p* = 0.001 (*d* = 0.62).

Victims of sexual femicide were younger (*M* = 34.16 years old; *SD* = 16.24) than victims of non-sexual femicide (*M* = 48.50 years old; *SD* = 20.53), *t*(360) = 5.98, *p* < 0.001 (*d* = 0.73). When comparing victims of sexual femicide with victims of rape (*M* = 30.40 years old; *SD* = 13.11), the former were slightly older than the latter, *t*(161) = 1.61, *p* = 0.06 (*d* = 0.25).

Victims of rape were also significantly younger than victims of non-sexual femicide, *t*(347) = 7.24, *p* = 0.001 (*d* = 0.94).

## Results

This study explored similarities and differences in looking first at sexual and non-sexual femicides. It also explored more specifically types of sexual femicides and explored whether certain factors were more likely involved in sexualized rather than in grievance femicide and vice versa. It finally compared cases in which rape was perpetrated without the escalation to femicide with cases in which it was, instead, a key part of the femicide dynamic.

Findings suggest that 500 victims were either killed (*n* = 365) or raped (*n* = 135) by 468 perpetrators. Overall, the great majority of the victims were known (67.7%; *n* = 335) to the perpetrator, while in 32.3% of cases (*n* = 160) the victims were unknown. In five cases it was not possible to establish whether the victims were known or unknown.

When the victim and the perpetrator knew each other, in 71.3% of cases (*n* = 239) they were in an intimate relationship, characterized by emotional, affective and sexual involvement. On average the intimate relationship was nearly 13 years long (*M* = 12.62; *SD* = 13.11; Range: 0.01–24.13). In 53.5% of cases (*n* = 174) the relationship was characterized by contentiousness.

### Comparing sexual femicide and non-sexual femicide

As shown in Table 3, the risk for women of being killed by a known perpetrator was lower in the case of sexual femicide (25.0%; *n* = 22) than in non-sexual femicide (82.4%; *n* = 224) (OR = 0.07; 95% CI = 0.04–0.13). When the victim knew the perpetrator, the intensity of the relationship with him (intimate vs. non-intimate, i.e., acquaintance/superficial) did not make any difference in how the woman was killed, either by a sexual femicide (72.7%; *n* = 16) or by a non-sexual femicide (70.5%; *n* = 158).

**TABLE 3:** Comparisons of sexual femicide versus non-sexual femicide versus rape.

Variables	Sexual femicide (A) *n* = 88	Non-sexual femicide (B) *n* = 275	Rape (C) *n* = 135	Odds Ratios (95% CI)	Odds Ratios (95% CI)	Odds Ratios (95% CI)
				A/B	A/C	B/C
**Type of victims**						
Known (1)/Unknown victims (0)	25.0% (*n* = 22)	82.4% (*n* = 224)	65.9% (*n* = 89)	0.07 (95% CI = 0.04–0.13)***	0.17 (95% CI = 0.10–0.31)***	2.41 (95% CI = 1.50–3.87)***
**Criminal careers of perpetrator**						
Previous criminal records: yes (1)/no (0)	34.5% (*n* = 30)	25.4% (*n* = 67)	62.2% (*n* = 84)	1.55 (95% CI = 0.92–2.61)	0.32 (95% CI = 0.18–0.56)***	0.21 (95% CI = 0.13–0.32)***
**Type of relationships**						
Prostitutes (1)/Non-prostitutes (0)	77.3% (*n* = 68)	10.5% (*n* = 29)	12.6% (*n* = 17)	28.84 (95% CI = 15.36–54.14)***	23.60 (95% CI = 11.58–48.10)***	0.82 (95% CI = 0.43–1.55)
Intimate (1)/Acquaintance—Superficial (0) Relationships	72.7% (*n* = 16)	70.5% (*n* = 158)	73.0% (*n* = 65)	1.11 (95% CI = 0.42–2.97)	0.99 (95% CI = 0.35–2.81)	0.88 (95% CI = 0.51–1.53)
**Characteristics of relationships**						
Duration of relationships: Long: 10 years plus (1)/Short-Medium: up to 9 years (0)	23.8% (*n* = 5)	55.7% (*n* = 102)	37.2% (*n* = 32)	0.25 (95% CI = 0.09–0.71)**	0.53 (95% CI = 0.18–1.58)	2.13 (95% CI = 1.26–3.59)**
Victim/Perpetrator Cohabitation: yes (1)/no (0)	4.5% (*n* = 3)	54.5% (*n* = 139)	44.4% (*n* = 60)	0.04 (95% CI = 0.01–0.13)***	0.06 (95% CI = 0.02–0.20)***	1.50 (95% CI = 0.99–2.28)^†^
Contentiousness: yes (1)/no (0)	15.6% (*n* = 10)	56.5% (*n* = 122)	31.1% (*n* = 42)	0.14 (95% CI = 0.07–0.30)***	0.41 (95% CI = 0.19–0.88)*	2.87 (95% CI = 1.83–4.52)***
Morbid jealousy: yes (1)/no (0)	10.9% (*n* = 5)	25.5% (*n* = 60)	25.3% (*n* = 24)	0.36 (95% CI = 0.13–0.94)*	0.36 (95% CI = 0.13–1.02)^†^	1.01 (95% CI = 0.59–1.75)
Stalking: yes (1)/no (0)	4.4% (*n* = 2)	12.0% (*n* = 28)	6.7% (*n* = 9)	0.34 (95% CI = 0.08–1.49)	0.65 (95% CI = 0.14–3.13)	1.90 (95% CI = 0.87–4.16)
Children involved (1)/not involved (0) in violence	3.3% (*n* = 1)	24.4% (*n* = 42)	46.9% (*n* = 30)	0.11 (95% CI = 0.01–0.81)*	0.04 (95% CI = 0.01–0.30)***	0.37 (95% CI = 0.20–0.67)**
**Motives for offending**						
Predatory—Antisociality (1)/Oppressive—Multiproblematic Relationships (0)	84.1% (*n* = 74)	34.0% (*n* = 90)	53.3% (*n* = 72)	10.28 (95% CI = 5.50–19.21)***	4.63 (95% CI = 2.38–8.98)***	0.45 (95% CI = 0.30–0.69)***
**Context of violence occurrence**						
Home (victim’s or perpetrator’s) (1)/Public Places (0)	31.0% (*n* = 27)	77.1% (*n* = 209)	65.2% (*n* = 88)	0.13 (95% CI = 0.08–0.23)***	0.24 (95% CI = 0.14–0.43)***	1.80 (95% CI = 1.14–2.83)*
**Aggravating factors**						
Overkill: yes (1)/no (0)	47.7% (*n* = 42)	42.9% (*n* = 118)	−	1.22 (95% CI = 0.75–1.97)	−	−
Substance use by the perpetrator: yes (1)/no (0)	15.9% (*n* = 14)	12.4% (*n* = 34)	53.3% (*n* = 72)	1.34 (95% CI = 0.68–2.63)	0.17 (95% CI = 0.09–0.32)***	0.12 (95% CI = 0.08–0.20)***
Weapons: improper (1)/proper (0)	66.7% (*n* = 58)	36.0% (*n* = 96)	38.9% (*n* = 21)	3.56 (95% CI = 2.14–5.94)***	3.14 (95% CI = 1.55–6.37)**	0.88 (95% CI = 0.48–1.61)
Body Exploitation: yes (1)/no (0)	53.4% (*n* = 47)	12.4% (*n* = 34)	3.7% (*n* = 5)	8.13 (95% CI = 4.68–14.11)***	29.81 (95% CI = 11.11–79.94)***	3.67 (95% CI = 1.40–9.61)**
Body Mutilation: yes (1)/no (0)	15.9% (*n* = 14)	1.8% (*n* = 5)	3.7% (*n* = 5)	10.22 (95% CI = 3.56–29.28)***	4.92 (95% CI = 1.70–14.20)**	0.48 (95% CI = 0.14–1.69)
Perpetrator’s Reaction after Crime: active (1)/passive (0)	94.8% (*n* = 73)	44.6% (*n* = 111)	94.1% (*n* = 127)	22.69 (95% CI = 8.04–64.00)***	1.15 (95% CI = 0.34–3.95)	0.05 (95% CI = 0.02–0.11)***

Rape in this study complies cases of rape ‘only’ and cases of rape within IPV. Percentages exclude missing values. Column percentage are shown. CI, confidence interval. ^†^*p* < 0.10; *p < 0.05; **p < 0.01; ***p < 0.001. See Supplementary material for a graphical representation of these results (Graph 1a, 1b, and 1c).

However, some differences emerged in the duration of the relationship. The relationship between the victim and perpetrator was shorter for sexual femicide than for non-sexual femicide^5^ (OR = 0.25; 95% CI = 0.09–0.71). The victim and perpetrator were less likely to live together (cohabitation) in the case of sexual femicide than in non-sexual femicide (OR = 0.04; 95% CI = 0.01–0.13), a finding that is consistent with the fact that sexual femicide was more likely to have occurred in public places rather than at home (OR = 0.13; 95% CI = 0.08–0.23). Looking at the quality of the relationship, contentiousness (OR = 0.14; 95% CI = 0.07–0.30) and morbid jealousy (OR = 0.36; 95% CI = 0.13–0.94) were less likely to influence sexual femicide than non-sexual femicide. No difference was found regarding stalking (see Table 3 and the Supplementary material for a detailed description of these results)^6^.

In sexual femicide, children were less likely to witness the violence than in non-sexual femicide, where children unfortunately witnessed the violence that led to their mothers’ femicide (OR = 0.11; 95% CI = 0.01–0.81).

When looking at the dynamic of violence, in sexual femicides it was more likely that the motives behind the violence were predatory or antisocial in comparison with non-sexual femicide (OR = 10.28; 95% CI = 2.14–5.94). It was more likely that sexual femicides occurred in a remote and secluded place in the outskirt of a city, in comparison with non-sexual femicides (OR = 0.13; 95% CI = 0.08–0.23). Perpetrators of sexual femicides more likely used an improper weapon (e.g., blunt objects) to kill in comparison with non-sexual femicides (OR = 3.56; 95% CI = 2.14–5.94), and also were eight times more likely to exploit the body of the victims even after the killing (OR = 8.13; 95% CI = 4.68–14.11). Furthermore, body mutilation was ten times higher in sexual femicide than in non-sexual femicide (OR = 10.22; 95% CI = 3.56–29.28). In sexual femicide perpetrators were more likely than in non-sexual femicide to escape after committing the crime (i.e., active reaction), in an attempt to avoid punishment (OR = 22.69; 95% CI = 8.04–64.00).

No difference was found in their criminal careers (e.g., previous criminal records) between sexual and non-sexual femicidal offenders (see Table 3 for details on these results).

Contrary to expectations, overkill *per se* was not significant in distinguishing sexual from non-sexual femicides. However, a separate analysis comparing known with unknown victims of femicide showed that the former (73.0%; *n* = 116) were at higher risk for overkill than the latter (54.7%; *n* = 130) (OR = 1.47; 95% CI = 0.94–2.32).

### The psychology of sexual femicides

Of the 365 victims of femicides included in this study, 88 were women killed because of sexual femicide. For two cases of femicides it was not possible to establish whether the femicide was of a sexual nature or not.

Twenty-five percent (*n* = 22) of these victims were known to the perpetrator. As mentioned before, among these known victims, 72.7% (*n* = 16) had an intimate relationship with the perpetrator, although this relationship rarely lasted more than a decade (23.8%; *n* = 5) and only in a few cases (4.5%; *n* = 3) involved cohabitation between them (see Endnote 3). In 10.9% of cases (*n* = 5) the relationship was characterized by morbid jealousy and in 15.6% of cases (*n* = 10) by contentiousness. In only one case did sexual femicide occur in the presence of the victim’s children (3.3%; *n* = 1).

As shown in Table 3, in 77.3% of sexual femicides the victims involved were prostitutes (*n* = 68) in comparison with 10.5% of prostitutes (*n* = 29) in non-sexual femicides (OR = 28.84; 95% CI = 15.36–54.14) and with 12.6% of prostitutes (*n* = 17) in rape (OR = 23.60; 95% CI = 11.58–48.10). No significant differences emerge when comparing non-sexual femicides with rape.

#### Are all sexual femicides of the same kind?

Looking specifically at the types of sexual femicide, we found that the vast majority of cases were sexualized femicides (76.1%; *n* = 67), while 23.9% (*n* = 21) were grievance femicides. Table 4 shows these results.

**TABLE 4:** Comparisons of grievance femicide versus sexualized femicide.

Variables	Sexual femicide		Odds Ratios (95% CI)
	Grievance femicide (A) *n* = 21	Sexualized femicide (B) *n* = 67	A/B
**Type of victims**			
Known (1)/Unknown victims (0)	68.2% (*n* = 15)	31.8% (*n* = 7)	0.05 (95% CI = 0.01–0.16)***
**Criminal careers of perpetrator**			
Previous criminal records: yes (1)/no (0)	42.9% (*n* = 9)	31.8% (*n* = 21)	0.62 (95% CI = 0.23–1.70)
**Type of relationships**			
Prostitutes (1)/Non-prostitutes (0)	42.9% (*n* = 9)	88.1% (*n* = 59)	9.83 (95% CI = 3.16–30.65)***
Intimate (1)/Acquaintance—Superficial (0) Relationships	86.7% (*n* = 13)	42.9% (*n* = 3)	0.16 (95% CI = 0.01–0.95)
**Characteristics of relationships**			
Duration of relationships: Long: 10 years plus (1)/Short-Medium: up to 9 years (0)	30.8% (*n* = 4)	12.5% (*n* = 1)	0.32 (95% CI = 0.03–3.56)
Victim/Perpetrator Cohabitation: yes (1)/no (0)	10.0% (*n* = 2)	2.2% (*n* = 1)	0.20 (95% CI = 0.02–2.35)
Contentiousness: yes (1)/no (0)	42.1% (*n* = 8)	4.4% (*n* = 2)	0.06 (95% CI = 0.01–0.35)***
Morbid jealousy: yes (1)/no (0)	22.2% (*n* = 4)	3.6% (*n* = 1)	0.13 (95% CI = 0.01–1.27)
Stalking: yes (1)/no (0)	11.8% (*n* = 2)	3.6% (*n* = 1)	0.28 (95% CI = 0.02–3.32)
Children involved (1)/not involved (0) in violence	28.6% (*n* = 6)	33.8% (*n* = 22)	1.80 (95% CI = 0.44–3.76)
**Motives for offending**			
Predatory/Antisociality (1)—Oppressive/Multiproblematic Relationships (0)	57.1% (*n* = 12)	92.5% (*n* = 62)	9.30 (95% CI = 2.65–32.65)***
**Context of violence occurrence**			
Home (victim’s or perpetrator’s) (1)/Public Places (0)	33.3% (*n* = 7)	30.3% (*n* = 20)	0.87 (95% CI = 0.31–2.48)
**Aggravating factors**			
Overkill: yes (1)/no (0)	57.1% (*n* = 12)	44.8% (*n* = 30)	0.61 (95% CI = 0.23–1.64)
Substance use by the perpetrator: yes (1)/no (0)	14.3% (*n* = 3)	16.4% (*n* = 11)	1.18 (95% CI = 0.30–4.70)
Weapons: improper (1) vs. proper (0)	57.1% (*n* = 12)	69.7% (*n* = 46)	1.73 (95% CI = 0.63–4.74)
Body Exploitation: yes (1)/no (0)	42.9% (*n* = 9)	56.7% (*n* = 38)	1.75 (95% CI = 0.65–4.70)
Body Mutilation: yes (1)/no (0)	0.05% (*n* = 1)	20.9% (*n* = 14)	5.28 (95% CI = 0.65–42.84)
Perpetrator’s Reaction after Crime: passive (0)/active (1)	85.7% (*n* = 18)	98.2% (*n* = 55)	9.17 (95% CI = 0.90–93.74)

Percentages exclude missing values. Percentages exclude missing values. Column percentage are shown. CI, confidence interval. ^†^*p* < 0.10; *p < 0.05; **p < 0.01; ***p < 0.001.

In 8 cases it was possible to identify an overlap between sexualized and grievance characteristics, albeit with a prevalence of characteristics typical of one or the other category. Although only involving a few cases, in 6 out of 8 cases the overlapping occurred when the victim was unknown and a prostitute.

When comparing sexualized with grievance femicides some significant differences emerged. Sexualized (90.9%; *n* = 60) in comparison with grievance (9.1%; *n* = 6) femicides more likely involved unknown victims (OR = 0.05; 95% CI = 0.01–0.16). When the victim and perpetrator knew each other, their relationship was characterized by a lower level of contentiousness in sexualized femicide (4.4%; *n* = 2) in comparison with grievance femicide (42.1%; *n* = 8) (OR = 0.06; 95% CI = 0.01–0.35). Prostitutes were more likely the victims of sexualized (88.1%; *n* = 59) than grievance femicides (42.9%; *n* = 9) (OR = 9.83; 95% CI = 3.16–30.65). Motives for sexualized femicides were mostly antisocial, characterized by sexual deviance, in comparison with grievance femicides in which the oppressive component of the relationship prevailed (OR = 9.30; 95% CI = 2.65–32.65).

No difference was found when compared the criminal careers of sexual and non-sexual femicidal offenders.

### Comparing sexual femicides and rape

When looking in more detail at all the forms of violence analyzed in this study, some interesting results emerge.

#### What do sexual femicides have in common with rape?

Sexual femicides were more likely to be committed by an unknown man in an anonymous setting, in comparison with rape, in which the perpetrator knew the victim (OR = 0.17; 95% CI = 0.10–0.31). In sexual femicide, the relationship between the victim and perpetrator was less likely characterized by contentiousness than in cases of rape (OR = 0.41; 95% CI = 0.19–0.88). The victim and perpetrator were less likely to live together in cases of sexual femicide compared with rape (OR = 0.06; 95% CI = 0.02–0.20). The intensity of the relationship between victim and perpetrator (i.e., intimacy) was not significantly different in the cases of sexual femicides and rape. The duration of their relationship, jealousy and stalking were not significant in differentiating when comparing sexual femicides and rape.

Typically, sexual femicide tended to occur more likely in secluded spaces (OR = 0.24; 95% CI = 0.14–0.43) in comparison with rape, that occurred more likely in either the victim’s or the perpetrator’s home. Substance abuse by the perpetrator around the time of the violence was more likely in rape than in sexual femicide (OR = 0.17; 95% CI = 0.09–0.32).

The use of improper weapons was more likely in sexual femicide than rape (OR = 3.14; 95% CI = 1.55–6.37). Body exploitation (OR = 29.81; 95% CI = 11.11–79.94) and body mutilation (OR = 4.92; 95% CI = 1.70–14.20) were more likely to occur in sexual femicide than in rape (see Table 3 for a detailed overview of the results). No difference was found in the types of reaction of the perpetrator after the crime.

### Comparing non-sexual femicides and rape

#### What do non-sexual femicides have in common with rape?

Knowing the perpetrator (OR = 2.41; 95% CI = 1.50–3.87), and being in a long relationship with him (OR = 2.13; 95% CI = 1.26–3.59) was more likely in non-sexual femicides rather than in rape (see note 3). When looking at the intensity of the relationship between victim and perpetrator (i.e., intimacy), no differences were found when comparing non-sexual femicides with rape. The risk for children witnessing the violence was strongly increased in those cases in which their mother was raped rather than killed (OR = 0.37; 95% CI = 0.20–0.67).

It was more likely that an oppressive relationship was behind the motives for non-sexual femicide rather than rape (OR = 0.45; 95% CI = 0.30–0.69). Non-sexual femicides were more likely committed at home in comparison with rape (OR = 1.80; 95% CI = 1.14–2.83). As expected, substance use by the perpetrator was more frequent in rape than in non-sexual femicide (OR = 0.12; 95% CI = 0.08–0.20).

The exploitation of the victim’s body was almost four times more likely in the case of non-sexual femicide rather than in rape (OR = 3.67; 95% CI = 1.40–9.61), while an active reaction of the perpetrator after the violence was more frequent in rape rather than in non-sexual femicide (OR = 0.05; 95% CI = 0.02–0.11). No difference was found for body mutilation. All these findings are shown in Table 3.

Looking at these findings, and in line with previous studies (Krantz and Garcia-Moreno, 2005; Fernandez, 2011) it is noticeable that rape is part of a form of combined violence against women because it emerges either as a form of sexual violence within IPV or as the prevalent form of violence (rape ‘only’).

### Looking for similarities and differences between sexual and non-sexual femicides and rape

These findings suggest that while sexual and non-sexual femicides share the killing of the victims, some aspects could make them more criminogenically closer to rape.

#### What do sexual femicides have in common with rape ‘only’ and rape within IPV?

As reported in Table 5, when comparing sexual femicide with either rape within IPV or rape ‘only’ some interesting findings emerged respectively. For instance, sexual femicides were more likely to differ from rape within IPV, because victims were more likely to be unknown while the victims of rape within IPV were more likely to be intimate, involved in an oppressive and contentious relationship with the perpetrator; often violence occurred in the presence of children and at home. Perpetrators of rape within IPV were more likely to have criminal records than for sexual femicide.

**TABLE 5:** Comparisons of sexual femicide and non-sexual femicide with rape within IPV and rape ‘only.’

Variables	Sexual femicide (A) *n* = 88	Rape within IPV (B) *n* = 63	Rape ‘only’ (C) *n* = 72	Odds Ratios (95% CI)	Odds Ratios (95% CI)	Non-sexual femicide (A) *n* = 275	Rape within IPV (B) *n* = 63	Rape ‘only’ (C) *n* = 72	Odds Ratios (95% CI)	Odds Ratios (95% CI)
				A/B	A/C				A/B	A/C
**Type of victims**										
Known (1)/Unknown victims (0)	25.0% (*n* = 22)	98.4% (*n* = 62)	37.5% (*n* = 27)	0.01 (95% CI = 0.00–0.04)***	0.56 (95% CI = 0.28–1.10)	82.4% (*n* = 224)	98.4% (*n* = 62)	37.5% (*n* = 27)	0.08 (95% CI = 0.01–0.56)**	7.78 (95% CI = 4.40–13.75)***
**Criminal careers of perpetrator**										
Prostitutes (1)/Non-prostitutes (0)	77.3% (*n* = 68)	7.9% (*n* = 5)	16.7% (*n* = 12)	39.44 (95% CI = 13.93–111.66)***	17.00 (95% CI = 7.67–37.66)***	10.5% (*n* = 29)	7.9% (*n* = 5)	16.7% (*n* = 12)	1.37 (95% CI = 0.51–3.69)	0.59 (95% CI = 0.28–1.22)
Previous criminal records: yes (1)/no (0)	34.5% (*n* = 30)	55.6% (*n* = 35)	68.1% (*n* = 49)	0.42 (95% CI = 0.22–0.82)*	0.25 (95% CI = 0.13–0.48)***	25.4% (*n* = 67)	55.6% (*n* = 35)	68.1% (*n* = 49)	0.27 (95% CI = 0.15–0.48)***	0.16 (95% CI = 0.09–0.28)***
**Type of relationships**										
Intimate (1)/Acquaintance—Superficial (0) Relationships	72.7% (*n* = 16)	93.5% (*n* = 58)	25.9% (*n* = 7)	0.18 (95% CI = 0.05–0.73)*	7.62 (95% CI = 2.13–27.22)**	70.5% (*n* = 158)	93.5% (*n* = 58)	25.9% (*n* = 7)	0.17 (95% CI = 0.06–0.47)***	6.84 (95% CI = 2.76–16.95)***
**Characteristics of relationships**										
Duration of relationships: Short-Medium: up to 9 years (0)/Long: 10 years plus (1)	23.8% (*n* = 5)	47.6% (*n* = 30)	8.7% (*n* = 2)	0.34 (95% CI = 0.11–1.05)^†^	3.28 (95% CI = 0.56–19.15)	55.7% (*n* = 102)	47.6% (*n* = 30)	8.7% (*n* = 2)	1.39 (95% CI = 0.78–2.46)	13.22 (95% CI = 3.01–58.05)***
Victim/Perpetrator Cohabitation: yes (1)/no (0)	4.5% (*n* = 3)	88.9% (*n* = 56)	5.6% (*n* = 4)	0.01 (95% CI = 0.00–0.02)***	0.81 (95% CI = 0.17–3.76)	54.5% (*n* = 139)	88.9% (*n* = 56)	5.6% (*n* = 4)	0.15 (95% CI = 0.07–0.34)***	20.37 (95% CI = 7.21–57.52)***
Contentiousness: yes (1)/no (0)	15.6% (*n* = 10)	63.5% (*n* = 40)	2.8% (*n* = 2)	0.11 (95% CI = 0.05–0.25)***	6.48 (95% CI = 1.36–30.82)*	56.5% (*n* = 122)	63.5% (*n* = 40)	2.8% (*n* = 2)	0.75 (95% CI = 0.42–1.33)	45.43 (95% CI = 10.86–190.02)***
Morbid jealousy: yes (1)/no (0)	10.9% (*n* = 5)	31.7% (*n* = 20)	12.5% (*n* = 4)	0.26 (95% CI = 0.09–0.76)*	0.85 (95% CI = 3.46)	25.5% (*n* = 60)	31.7% (*n* = 20)	12.5% (*n* = 4)	0.74 (95% CI = 0.40–1.35)	2.40 (95% CI = 0.69–13.73)
Stalking: yes (1)/no (0)	4.4% (*n* = 2)	14.3% (*n* = 9)	1.4% (*n* = 1)	3.30 (95% CI = 0.29–37.52)	0.37 (95% CI = 0.30–0.47)	12.0% (*n* = 28)	14.3% (*n* = 9)	1.4% (*n* = 1)	0.82 (95% CI = 0.36–1.83)	0.11 (95%CI = 0.01–0.77)**
Children involved (1)/not involved (0) in violence	3.3% (*n* = 1)	65.1% (*n* = 28)	9.5% (*n* = 2)	0.02 (95% CI = 0.00–0.15)***	0.33 (95% CI = 0.03–3.87)	24.4% (*n* = 42)	65.1% (*n* = 28)	9.5% (*n* = 2)	0.17 (95% CI = 0.08–0.36)***	3.07 (95% CI = 0.42–1.33)
**Motives for offending**										
Predatory/Antisociality (1)/Oppressive—Multiproblematic Relationships (0)	84.1% (*n* = 74)	12.7% (*n* = 8)	88.9% (*n* = 64)	36.34 (95% CI = 14.25–92.67)***	0.66 (95% CI = 0.26–1.68)	34.0% (*n* = 90)	12.7% (*n* = 8)	88.9% (*n* = 64)	3.54 (95% CI = 1.61–7.74)**	0.06 (95% CI = 0.03–0.14)***
**Context of violence occurrence**										
Home (victim’s or perpetrator’s) (1)/Public Places (0)	31.0% (*n* = 27)	90.5% (*n* = 57)	43.1% (*n* = 31)	0.05 (95% CI = 0.02–0.12)***	0.60 (95% CI = 0.31–1.14)	77.1% (*n* = 209)	90.5% (*n* = 57)	43.1% (*n* = 31)	0.36 (95% CI = 0.15–0.86)*	4.46 (95% CI = 2.58–7.70)***
**Aggravating factors**										
Substance use by the perpetrator: yes (1)/no (0)	15.9% (*n* = 14)	54.0% (*n* = 34)	52.8% (*n* = 38)	0.16 (95% CI = 0.08–0.34)***	0.17 (95% CI = 0.08–0.35)***	12.4% (*n* = 34)	54.0% (*n* = 34)	52.8% (*n* = 38)	0.12 (95% CI = 0.07–0.22)***	0.13 (95% CI = 0.07–0.23)***
Weapons: improper (1)/proper (0)	66.7% (*n* = 58)	45.2% (*n* = 14)	30.4% (*n* = 7)	2.43 (95% CI = 1.05–5.60)^†^	4.57 (95% CI = 1.69–12.35)**	36.0% (*n* = 96)	45.2% (*n* = 14)	30.4% (*n* = 7)	0.68 (95% CI = 0.32–1.44)	1.28 (95% CI = 0.51–3.23)
Body Exploitation: yes (1)/no (0)	53.4% (*n* = 47)	6.3% (*n* = 4)	1.4% (*n* = 1)	16.91 (95% CI = 5.65–50.59)***	81.39 (95% CI = 10.82–612.12)***	12.4% (*n* = 34)	6.3% (*n* = 4)	1.4% (*n* = 1)	2.08 (95% CI = 0.71–6.09)	10.02 (95% CI = 1.35–74.47)**
Body Mutilation: yes (1)/no (0)	15.9% (*n* = 14)	6.3% (*n* = 4)	1.4% (*n* = 1)	2.79 (95% CI = 0.87–8.93)	13.43 (95% CI = 1.72–104.84)**	1.8% (*n* = 5)	6.3% (*n* = 4)	1.4% (*n* = 1)	0.27 (95% CI = 0.07–1.05)	1.32 (95% CI = 0.15–11.43)
Perpetrator’s Reaction after Crime: passive (0)/active (1)	94.8% (*n* = 73)	96.8% (*n* = 61)	91.7% (*n* = 66)	0.60 (95% CI = 0.11–3.38)	1.66 (95% CI = 0.45–6.14)	44.6% (*n* = 111)	96.8% (*n* = 61)	91.7% (*n* = 66)	0.03 (95% CI = 0.01–0.11)***	0.07 (95% CI = 0.03–0.18)***

Rape in this study is distinguished in cases of rape ‘only’ and cases of rape within IPV. Percentages exclude missing values. Column percentage are shown. CI, confidence interval. ^†^*p* < 0.10; *p < 0.05; **p < 0.01; ***p < 0.001.

Table 5 reports all results of this comparative analysis.

On the other hand, sexual femicide and rape ‘only’ involved mostly unknown victims. They were more likely to occur in public places and mainly for predatory and antisocial motives. In those cases of rape ‘only,’ it was more likely that the perpetrator was under the effect of alcohol or drugs. These offenders had also more previous criminal records in comparison with sexual femicidal offenders.

#### What do non-sexual femicides have in common with rape ‘only’ and rape within IPV?

Non-sexual femicides were similar to rape within IPV because both involved victims and perpetrators in a longer relationship, characterized by high level of contentiousness between them, and morbid jealousy. However, there were some differences when looking at the motives for violence which were more likely to be predatory and antisocial in non-sexual femicide than in rape within IPV. Perpetrators of rape within IPV were more likely to report previous criminal records. It was more likely that rape within IPV occurred at home and that abuse of substance was involved (see Table 5 for the details regarding these results).

Non-sexual femicides differently from rape ‘only’ involved known victims and were more likely to have occurred within an oppressive and multiproblematic relationship. Body exploitation was more likely in the cases of non-sexual femicide suggesting the use of some forms of torture during the violence.

It is interesting to note that perpetrators of non-sexual femicide were more likely to admit the crime in comparison with perpetrators of both rape within IPV and rape ‘only,’ who more likely either denied or escaped from the crime scene (see Table 5).

## Discussion

This study examines cases of violence against women in northern Italy, focusing on sexual and non-sexual femicides and comparing them with rape (rape within IPV and rape ‘only’) (see Figure 1 for a summary of these forms of violence). Rape by itself or in combination with IPV or femicide, i.e., sexual femicide, means the violation of the most intimate dimension of a person: her own sexuality.

The results presented here, in line with other research findings (Campbell et al., 2003; Veggi et al., 2021), show that these types of violence against women reveal interesting similarities regarding types of victims, and the turbulent, oppressive, and domineering relationships with their perpetrators.

Sexual femicides exhibited a specific pattern in terms of victims (more likely to be unknown), of motives (more likely to be antisocial and sexually deviant), of characteristics of the crime dynamic (more likely to occur in secluded locations), and of perpetrators who were more likely to use improper weapons and to escape from the crime scene compared to non-sexual femicides. Sexual femicides also shared some similarities with rape, particularly regarding victim only known superficially or only involved in a short-term relationship with the perpetrator. Women who are victims of sexual femicide often experience a prolonged pattern of abuse and torture, are often left unclothed after being raped and mutilated. Whether or not they know their killer influences how and why they become the target of such extreme violence.

### Sexual versus non-sexual femicides

Sexual femicide was seen in this study as an extreme form of control and domination in which sex was used to degrade the woman to the point of death. The manifestation of violence was characterized by a specific pattern. Victims of sexual femicide were indeed more likely to be unknown and to be killed in a remote public place, with an improper weapon. Results also show that victims who engaged in prostitution were at a higher risk than other women of being victim of sexual femicides. Prostitutes were more likely victims of a sexualized femicide.

As emerged in other international studies (Regoeczi and Miethe, 2003; Zara et al., 2021), knowing or not knowing the perpetrator could make a difference in the way the woman was killed. These findings are consistent with statements in the literature (Meloy, 2000) that sexual femicides tend to be characterized by further exploitation of the victim’s body and mutilation: an ultimate expression of control, deviant sexuality, and anger toward women are seen as the main incentives for this form of lethal violence.

However, most women in this study were killed by a man they knew, albeit with different levels of involvement with him and duration of the relationship (see Table 1 for a description of the variables involved). As shown in Table 3, the type of relationship between victim and perpetrator could impact on how violence was acted out and on its intensity. Known victims were more exposed to non-sexual femicide, which more likely took place at home, in the context of a domestic dispute, and in the presence of their children, as other studies suggest (see Matias et al., 2020). It was not unusual that in non-sexual femicide the perpetrator either confessed the killing or committed suicide soon after the femicide.

It is Geberth (2018) who suggested that a certain type of abusive personality, under certain situational settings, can escalate to the worst because “an enraged lover or spouse who is acting under extreme emotional circumstances is capable of anything” (p. 452 as cited in Stefańska et al., 2021, p. 16). In non-sexual femicides, the relationship between victim and perpetrator was more likely to be characterized by contentiousness, suggesting a prolonged involvement in an oppressive climate of emotional tension, where a destructive, strained and turbulent relationship between them was, often, the trigger to femicide. This is why this form of violence against women is considered preventable (Jung and Stewart, 2019): early intervention could prevent the lethal escalation of IPV to femicide.

### Rape within IPV and rape ‘only’: A brief comparison with non-sexual and sexual femicides

In this research, rape emerged as part of a pattern of intimate partner violence (rape within IPV) or as a sexual crime (rape ‘only’). Known victims were more likely victims of intimate partner violence within which sexual violence clustered together abuse, control, domination, humiliation, making it a ‘composite’ form of violence (Table 5 summarizes these results).

Men strive for control, and rape, especially when occurred within IPV, can be one of the most disempowering experience for the woman. The more the woman is deprived of her choice and consent, the stronger the man’s control becomes. Victims of rape within IPV seem to share more characteristics with the victims of non-sexual femicide: the nature and duration of the relationship between victim and perpetrator could explain most of the similarities between these apparently different forms of violence against women (see Table 5 which summarizes these results).

As mentioned previously, and sustained by other studies, non-sexual femicide and rape within IPV are in line with the concept of proprietariness in which the woman is entitled to exist to the extent her partner allows her to do so (Gino et al., 2019; Spencer and Stith, 2020). This was evident in many cases of rape within IPV such as the one described below.

“A woman (38 years old) decided to leave her abusive and violent partner after countless episodes of physical, sexual, psychological, and economic violence in a relationship that lasted more than 10 years. For him, it was unacceptable that she tried to break up with him, and he demanded to get back the dentures he had paid for. The woman lost all her teeth in one of the abusive incidents she experienced in her relationship with him. She remained with him for many years after this incident.”

Sexual femicides tend to be an extreme form of using the woman as a sexual commodity.

Results show that in this study sexual femicides took different forms of sexualized and grievance, depending on the prevalent role of mostly sex or anger in the escalation into femicide, and on the relationship between perpetrator and victim.

The *use, consume and dispose script* is mostly featured in these types of sexual femicide because “what rape is to others is normal to *prostitutes*” (Farley et al., 2005, p. 254, *italics* added, as cited in Zara et al., 2021, p. 20). The fact that a significant proportion of victims of sexual femicides in this study were prostitutes is supported in the literature (e.g., Salfati, 2000; Salfati et al., 2008).

Victims were more likely to be unknown, to be abused and killed for antisocial and sexually deviant motives, and in secluded places. However, while in the case of sexual femicide it was more likely that improper weapons were used by the killer, in the cases of rape ‘only’ substance abuse by the perpetrator was a factor that enhanced the disinhibition by which the sexualized anger was manifested.

When looking at victims of rape, especially of rape ‘only,’ our findings suggest that they shared similar characteristics with victims of sexualized femicides who are more likely prostitutes. Similarities and differences are synthesized in Tables 3, 5.

### Grievance and sexualized femicides

As shown in Table 4, victims of grievance femicides were more likely to know their perpetrators, and less likely to be prostitutes. What seems to have prevailed for these types of sexual femicidal offenders were angry cognitive schemas that might have promoted the excessively aggressive response in what might have been initially a consensual sexual situation (Stefańska et al., 2015). In other words, as suggested in the literature (Beech et al., 2005), grievance femicides were driven by the preoccupation and anxiety of losing contact with the woman (e.g., fear of being deserted), high emotionality, and angry rumination.

On the other hand, sexualized femicides involved more frequently unknown victims, although perpetrators may have targeted specific victims (e.g., prostitutes). Sexualized femicides were likely to be driven by predatory schema in which the victim was dehumanized and her body made the target of further exploiting actions. This is in line with clinical studies that suggest that victims of sexualized femicides are used as a sexual commodity to satisfy sexual, sadistic, and pervert urges (Meloy, 1997a). Such exploiting behavior was found to be more representative of sexual femicides than both non-sexual femicides and sexual violence.

### Not dead enough: Body exploitation and mutilation

The presence of body exploitation and body mutilation of victims was examined and then compared with both sexual and non-sexual femicide, and also compared with rape. In line with other studies (Beech et al., 2005; Stefańska et al., 2015), these findings showed that the sexual factor in sexual femicides depended on whether the victims knew the perpetrators or not, and on the victims themselves. For instance, those victims whose body was exploited after their killing were more likely to be prostitutes. Prostitutes are over-exposed to the risk of workplace violence by their clients and tend to be killed more heinously than women who are not prostitutes, regardless of the degree of knowledge of the perpetrator (Marco Francia, 2021; Zara et al., 2021).

### Stalking

Even though not directly explored, stalking occurred in only about 10% of the sample. It is possible that many stalking behaviors went undetected or not reported by the victims (Miller, 2012). Results show that stalking was more likely in cases of rape within IPV, suggesting that obsessive pursuit of intimacy is reinforced by a partner who is perceived as attainable the more the perpetrator attempts to control and dominate her. These preliminary findings are in line with international literature (Douglas and Dutton, 2001; Fox et al., 2011) that indicate that stalking and IPV share many similar dimensions because both crimes are characterized by unwanted, harassing, intrusive and frightening and/or intimidating behaviors.

Stalking has rarely been examined in studies of sexual and non-sexual femicide, and the extent of the association is not well understood (Campbell et al., 2007; Stefańska et al., 2021). Further specific research on stalking behavior in cases of femicide and rape deserves attention, as early identification of conditions that promote and reinforce the pursuit of intimacy may help prevent femicide and prevent the continuation of violence.

## Limitations of the study

These findings are not without limitations. All femicide data were retrospective, and it was not possible to gather first-hand information from family members about the quality of the relationship between victim and perpetrator, and from perpetrators about the motives behind the killing. The evidence gathered explains only part of the dynamics of the intimate partner violence that fostered the femicide. Furthermore, it was impossible, with these data, to reconstruct with preciseness the victimogenic factors that interacted with other factors to escalate into sexual and non-sexual femicides, and that distinguished them from those victimogenic factors involved in rape.

Information on the victims were gathered through clinical and forensic reports so it was not possible to examine directly and in detail the conditions in which the victims were in before the crime (e.g., anxiety and preoccupation over their lives). Specific information about victims could be helpful for organizing preventive interventions.

We were unable to explore further the sexual nature of femicides because the information gathered were exclusively based on the forensic pathology reports, while access to the family members of the victims or to the offenders was not possible. Needless to say that getting in contact with the family members of the victims or perpetrators was beyond the scope of this study. Research findings (Beech et al., 2005; Stefańska et al., 2015) showed that deviant sexual and sadistic fantasies are an important factor in the characterization of sexual femicides. However, because analyses were based on the information presents in the files available, in this study it was not possible to assess the impact of deviant fantasies upon femicides.

Despite these limitations, this study contributes to understanding similarities and differences in these forms of violence against women. Non-sexual femicides and rape within IPV have in common aspects related to known victims with whom the perpetrators had an intimate relationship. The relationship between victim and perpetrator can make a difference in how, where and for how long the abuse is endorsed before transforming itself into the physical death of the woman.

Sexual femicides and rape ‘only’ are likely to involve unknown victims. Sex was the common denominator of these types of violence against women.

## Conclusion

While the ultimate goal of science and governments is to prevent all forms of violence against women, their intermediate goal should be to prevent violence from worsening to the point of killing. The fact that nearly a quarter of perpetrators had prior criminal records is an indication that violence against women in all its forms is a crime characterized by significant persistence over time, and it is possible that some violent incidents went unreported, contributing to the dark number that features in these different forms of violence against women. Governments should always rely on evidence-based research in order to make it possible for every woman to *begin again* in life, and not in death.

Given that ‘until death all is life,’ to paraphrase Paley (1999), every woman deserves the open destiny of life and should be assured that human rights are fully respected and endorsed because violence against women is a crime that victimizes all people, not just women.

## Data availability statement

The dataset involves sensitive data (e.g., criminal records, forensic files) and only in particular circumstances could be made it available under the authorization of authorities. Requests to access the datasets should be directed to GZ, georgia.zara@unito.it.

## Ethics statement

The studies involving human participants were reviewed and approved by in order to meet all the ethical standards, the researchers followed all possible procedures to ensure confidentiality, fair treatment of data and information, and to guarantee, at each stage of the research, that the material was treated with respect and discretion. The research protocol was organized according to The Italian Data Protection Authority Act nr. 9/2016 (art. 1 and 2: application and scientific research purposes; art. 4: cases of impossibility to inform the participants, e.g., deceased people), to The Code of Ethics of the World Medical Association (Declaration of Helsinki) for experiments involving humans (2013), and to the recent General Data Protection Regulation (GDPR) (2018). It was carried out in line with the Italian and the EU code of human research ethics and conduct in psychology, forensic pathology and legal medicine. Both research projects were approved by the Bioethics Committee of the University of Turin (respectively for the femicide study with protocol nr. 191414/2018; for the sex offending project with protocol nr. 6494/2018). The patients/participants provided their written informed consent to participate in this study.

## Author contributions

GZ, SG, SV, and FF conceived, planned, and organized the study, drafted the article, and interpreted the results. GZ and SV attained and coded the data and critically revised the article. GZ designed the study and analyzed the data. All authors contributed to the article and approved the submitted version.

## Footnotes

1 For a more comprehensive view of statistics see also: https://worldpopulationreview.com/country-rankings/rape-statistics-by-country.

2 Considering that the victims were exclusively women and the perpetrators exclusively men, we are confident that it is appropriate to speak of sexual and non-sexual femicides when referring to this study, even though Skott et al. (2018) used the general term “homicide.”

3 Data on femicide were collected at the Institute of Legal Medicine and at the Archive of the Morgue of Turin and they are part of a longitudinal research project on VaW (Zara and Gino, 2018). Data on rape are from the SORAT (Sex Offending Risk Assessment and Treatment) project (Zara, 2018). All data were anonymized and made unidentifiable; data were also numerically coded for statistical purposes.

4 According to Cohen (1992), values of effect size are interpreted as follows: 0.20 = small effect. 0.50 = medium effect. 0.80 = large effect.

5 On average the length of relationship between the victim and perpetrator was 6.02 years long (*SD* = 8.07) in the case of sexual femicide, and 15.06 years long (*SD* = 14.08) for non-sexual femicide, *t*(195) = 2.53, *p* = 0.01, (*d* = 0.66). On average the length of relationship between the victim and perpetrator was longer in the case of femicide, than for rape (*M* = 9.62; *SD* = 10.61), *t*(263) = 3.15, *p* = 0.001, (*d* = 0.42). The length of relationship between the victim and perpetrator was slightly shorter (*M* = 6.02; *SD* = 8.07) in the case of sexual femicide, than for rape, *t*(98) = −1.29, *p* = 0.01, (*d* = 0.35).

6 A graphical representation of the comparative results of sexual vs. non-sexual femicides vs. rape can be found in the Supplementary material (see Graph 1a, 1b, and 1c).
