# Supplementary material for: Sexual femicide, non-sexual femicide and rape: Where do the differences lie? A continuum in a pattern of violence against women
Source: Front Psychol. 2022 Nov 1;13:957327. doi: 10.3389/fpsyg.2022.957327 (PMC9664082; doi:10.3389/fpsyg.2022.957327)
Supplement: Supplementary file 1 [file Data_Sheet_1.PDF]

Graph 1a. Graphical representation of results reported in Table 3 Comparisons of Sexual Femicide *versus* Nonsexual Femicide.

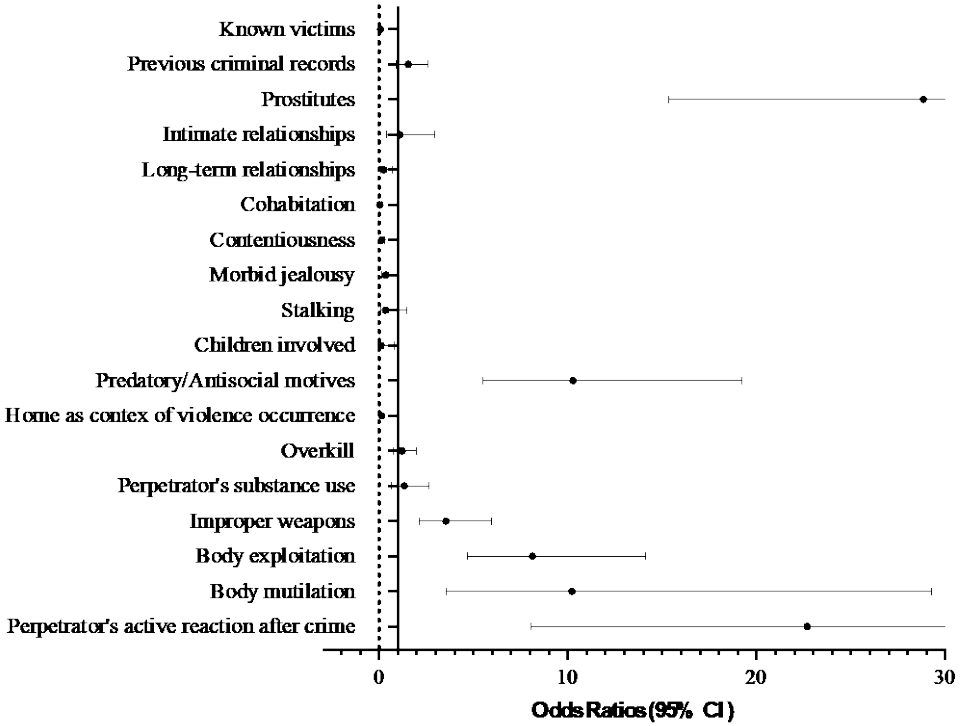

ODDS RATIOS (XY, 95% CI)
.07***
1.55
28.84***
1.11
.25**
.04***
.14***
.36*
.34
.11*
10.28***
.13***
1.22
1.34
3.56***
8.13***
10.22***
22.69***

Note: This graph 1a. shows a graphical representation of results summarized in Table 3 in which sexual femicide is compared specifically with nonsexual femicide on a set of variables.

Graph 1b. Graphical representation of results reported in Table 3 Comparisons of Sexual Femicide *versus* Rape.

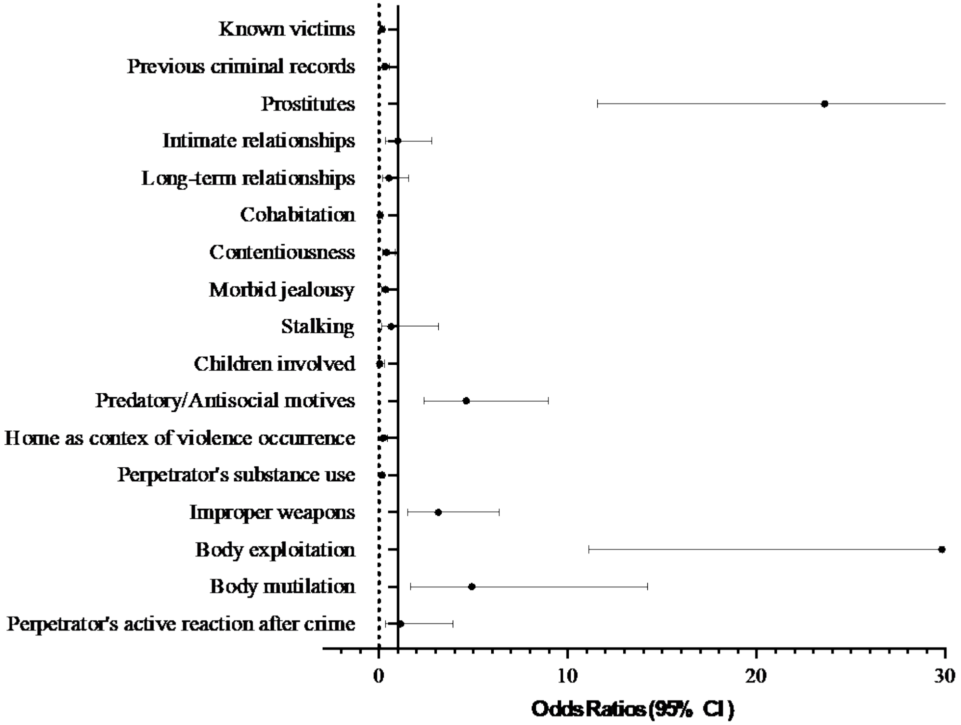

ODDS RATIOS (XY, 95% CI)
.17***
.32***
23.60***
.99
.53
.06***
.41*
.36[†]
.65
.04***
4.63***
.24***
.17***
3.14**
29.81***
4.92**
1.15

Note: This graph 1b. shows a graphical representation of results summarized in Table 3 in which sexual femicide is compared specifically with rape on a set of variables.

Graph 1c. Graphical representation of results reported in Table 3 Comparisons of Nonsexual Femicide *versus* Rape.

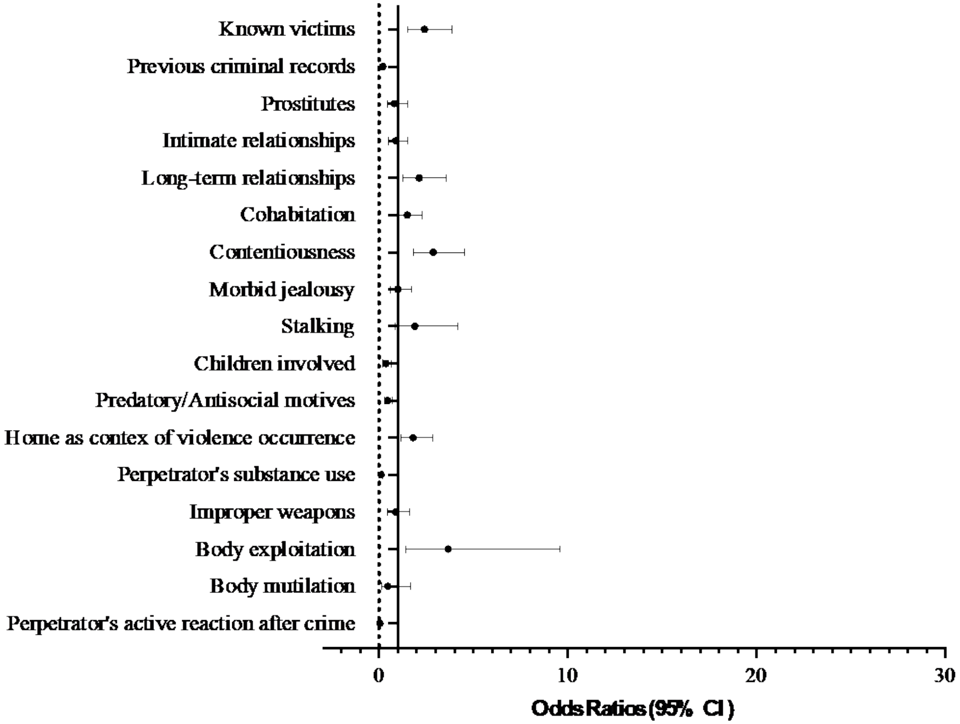

ODDS RATIOS (XY, 95% CI)
2.41***
.21***
.82
.88
2.13**
1.50†
2.87***
1.01
1.90
.37**
.45***
1.80*
.12***
.88
3.67**
.48
.05***

Note: This graph 1c. shows a graphical representation of results summarized in Table 3 in which nonsexual femicide is compared specifically with rape on a set of variables.
